# The Relationship between Handedness and Mathematics Is Non-linear and Is Moderated by Gender, Age, and Type of Task

**DOI:** 10.3389/fpsyg.2017.00948

**Published:** 2017-06-09

**Authors:** Giovanni Sala, Michela Signorelli, Giulia Barsuola, Martina Bolognese, Fernand Gobet

**Affiliations:** ^1^Department of Psychological Sciences, University of LiverpoolLiverpool, United Kingdom; ^2^Department of Oncology and Hemato-Oncology, University of MilanMilan, Italy; ^3^Maastricht UniversityMaastricht, Netherlands

**Keywords:** handedness, mathematics, lateralization, non-linearity, cognitive ability

## Abstract

The relationship between handedness and mathematical ability is still highly controversial. While some researchers have claimed that left-handers are gifted in mathematics and strong right-handers perform the worst in mathematical tasks, others have more recently proposed that mixed-handers are the most disadvantaged group. However, the studies in the field differ with regard to the ages and the gender of the participants, and the type of mathematical ability assessed. To disentangle these discrepancies, we conducted five studies in several Italian schools (total participants: *N* = 2,314), involving students of different ages (six to seventeen) and a range of mathematical tasks (e.g., arithmetic and reasoning). The results show that (a) linear and quadratic functions are insufficient for capturing the link between handedness and mathematical ability; (b) the percentage of variance in mathematics scores explained by handedness was larger than in previous studies (between 3 and 10% vs. 1%), and (c) the effect of handedness on mathematical ability depended on age, type of mathematical tasks, and gender. In accordance with previous research, handedness does represent a correlate of achievement in mathematics, but the shape of this relationship is more complicated than has been argued so far.

## Introduction

Students' achievement in mathematics is a matter of high practical relevance. Mathematical skill is necessary to major in Science, Technology, Engineering, and Mathematics (STEM) subjects, and therefore to attain STEM jobs. The job market requires worldwide more graduates in STEM subjects than in other disciplines (e.g., humanities, social sciences) and has also become increasingly more competitive (Halpern et al., 2007). For this reason, the cognitive and biological correlates of mathematical ability have been the object of extensive debate (e.g., Deary et al., 2007; Rohde and Thompson, 2007; Wai et al., 2009; Lubinski, 2010; Peng et al., 2016). One of these correlates is handedness.

Handedness is a manifestation of the lateralization of human brain function, and consequently, hand preference is believed to affect human overall cognitive skills (McManus, 2002). However, in spite of much research carried out on this topic, the shape of this relationship is still highly controversial.

The effect of handedness on mathematical ability have been a matter of interest too (e.g., Annett and Kilshaw, 1982; Benbow, 1986, 1987; Annett and Manning, 1990; Crow et al., 1998; Cheyne et al., 2010). However, no distinct pattern of results has emerged from the research addressing this topic. For example, while some studies considered left-handedness as a sign of giftedness in mathematics (e.g., Benbow, 1986), others found that left-handers performed slightly worse than right-handers on measures of mathematical ability (e.g., Johnston et al., 2013). The unclear relationship between handedness and mathematics reflects the discrepancies between the models relating handedness to cognitive abilities. In fact, according to Nicholls et al. (2010), four main models linking handedness to human cognition have been proposed. Each of these models makes different predictions about how handedness affects mathematical ability.

One of the most influential models linking handedness, cognition, and mathematical ability is Annett's (1985, 2002) *right shift theory*. According to this theory, most people inherit the so-called “right-shift factor,” which is a dominant allele (RS+) that predisposes them to be both right-handed and left-hemisphere dominant for language. Whoever inherits this allele has a good probability of being right-handed (see Corballis, 1997, for a review). While people with a heterozygous genotype (RS±) are mostly moderately right-handed (i.e., they do not show an exclusive preference for the right hand in experimental or daily life tasks), and benefit from a balanced cognitive profile, those who inherit a homozygous genotype for the RS+ are mostly strongly right-handed and may suffer from a deficit in spatial ability, because of the costs the RS+ allele to the right-hemisphere. Finally, in people who do not inherit the right-shift factor (i.e., homozygous for the RS– allele), handedness is determined by random factors (active during fetal development) and by environmental pressure on hand use. In this situation, a person ends up being randomly either right- or left-handed, and may suffer from a deficit specific to language ability.

Annett's right-shift theory thus predicts a general cognitive advantage for moderate right-handers. In line with this hypothesis, Annett (1992) found an advantage in spatial ability for moderate right-handers in a sample of 14–15-year-olds. Further support for this hypothesis was also provided by Casey's (1995, 1996a) studies, which found an advantage for moderate right-handers in general intelligence in a sample of primary school children, and mental rotation ability in a sample of female college students, respectively. However, some studies failed to replicate these outcomes (e.g., McManus et al., 1993; Cerone and McKeever, 1999). Finally, a more recent study (Nicholls et al., 2010) showed that moderate right-handers performed slightly better than the rest of the sample in a test measuring several cognitive skills such as attention, executive functions, language ability, and memory. Interestingly, in that study, both strong right- and left-handers achieved the worst results, thus showing a quadratic relationship between hand skill and test scores.

Concerning mathematical ability, Annett's right shift theory predicts a disadvantage for strongly right-handed individuals (Peters, 1991) and an inverse linear relationship between dextrality. Since mathematical ability relies to a large extent on spatial ability (Wai et al., 2009; Lubinski, 2010), those who inherit the homozygous dominant genotype are more likely to perform poorly in mathematics (Annett and Manning, 1990).

Following another line of research, Benbow (1986) claimed that left-handedness is a predictor of mathematical precociousness in young students. In that study, it was found that the rates of left-handers among students talented in mathematics were much greater than among the general population. Benbow (1986) found that the frequency of left-handedness among gifted students was significantly higher than in the general population. Moreover, this alleged superiority of left-handers seems to occur mostly in males and when mathematical ability is assessed with tasks involving reasoning (e.g., mathematical problems; Benbow, 1988).

Benbow's hypothesis—i.e., left-handers tend to be overrepresented among gifted students—is based on the fact that left-handers are more likely^1^ to have a more developed right-hemisphere (Geschwind and Behan, 1984; Geschwind and Galaburda, 1987; O'Boyle and Benbow, 1990), which is involved in processes related to mathematical ability—such as spatial reasoning (Ganley and Vasilyeva, 2011) and mental rotation ability (O'Boyle et al., 2005; Hoppe et al., 2012), and a larger corpus callosum (Witelson, 1985; Beaton, 1997). This may foster interhemispheric connectivity and bi-hemispheric representation of cognitive functions (Benbow, 1986), with positive effects on left-handed individuals' intellectual skills, such as verbal reasoning (Halpern et al., 1998) and verbal fluency (Hines et al., 1992), episodic memory (Christman and Propper, 2001), intelligence among gifted children (Hicks and Dusek, 1980), and spatial abilities (Casey et al., 1992; Reio et al., 2004). In addition, the relationship between left-handedness and giftedness seems to occur in several domains. For example, left-handedness appears to be more common among gifted musicians (Kopiez et al., 2006), chess players (Gobet and Campitelli, 2007; Oremosu et al., 2011), artists (Preti and Vellante, 2007), and mathematicians (Annett and Kilshaw, 1982).

Recently, however, the idea that left-handedness is a predictor of superior intellectual ability has been challenged. Several authors have claimed that left-handedness is not related to any advantage in cognitive skills, and may even exert detrimental effects on general cognitive abilities and hence academic achievement. Left-handedness may be caused by left-hemisphere damage occurring pre- or peri-natally (Satz et al., 1985), and consequently, a portion of left-handed individuals may suffer from an overall cognitive deficit. In line with this hypothesis, Johnston et al. (2009) found that left-handed children slightly underperformed in a series of developmental measures, compared to right-handers. Also, two recent meta-analyses (Papadatou-Pastou and Tomprou, 2015; Somers et al., 2015) reported that left-handers were over-represented among intellectually challenged individuals and did slightly worse in spatial ability tasks, respectively. Consistent with these results, Johnston et al. (2013) found that left-handers underperformed in mathematical ability in a sample of children aged 5–14.

The last theory drawing a causal link from handedness to mathematical ability through cognition is the *hemispheric indecision* hypothesis (Crow et al., 1998). This theory focuses on the importance of handedness as a continuous variable, in opposition to the dichotomy left/right. The lateralization of brain function seems to be an advantage from an evolutionary perspective, because it obviates functional redundancy, and therefore makes neural processing run more efficiently (Gutwinski et al., 2011). Thus, the most decisive factor is *how much* a person is right or left-handed because a weak lateralization may be associated with a delay in development (Orton, 1937; Zangwill, 1960; Bishop, 1990).

This hypothesis has recently received empirical support. Crow et al. (1998) found that the tendency to show an equal skill for right and left hand in a square-checking task predicted deficits in verbal, non-verbal, and mathematical abilities in a sample of 11-year-old children. Peters et al. (2006) reported that individuals with no preferred hand in writing had the lowest performance in mental rotation ability. Corballis et al. (2008) observed that children who had no hand preference for writing performed significantly worse than right- and left-handers in several tasks including arithmetic, memory, and reasoning. Finally, Cheyne et al. (2010) found similar results in a large sample of 11-year-old children.

## Materials and methods

### The present study

The research that has been carried out on the effects of handedness on mathematical ability depicts an intricate tapestry. The outcomes of the studies are, at least partially, contradictory, and it is hard to infer a definitive conclusion from them. However, research also suggests that one reason for the discrepant findings may be methodological inconsistencies. Studies differed with regard to (a) how participants were categorized according to handedness (e.g., right-/left-handers, right-/left-/mixed-handers, non-right-/right-handers), which causes difficulties for comparing the outcomes between studies (Casey, 1996b; Cerone and McKeever, 1999; Li et al., 2003; Nicholls et al., 2010); (b) ages and educational levels (e.g., primary school children, middle- and high-school students, adults) of the participants; and (c) the specific mathematical abilities assessed (e.g., simple arithmetic or problem-solving).

The aim of this study was to reconcile the discrepancies observed in previous research in the field. First, we used a continuous measured of handedness without using any categorization. Second, we systematically manipulated the age of the participants and the mathematical tasks to evaluate the effect of these moderating variables on the relationship between handedness and performance on tests of mathematics.

### A theoretical challenge: the use of quartic functions

Along with the abovementioned methodological issues, another aspect of the research in the field may be a critical limitation. While the four theories we have reviewed differ in important ways, a common characteristic is that they consider the link between handedness and mathematical ability as dichotomous (e.g., left-handers vs. than right-handers), or, when a continuous measure of handedness is used, linear or quadratic. However, no study, to the best of our knowledge, has investigated the possibility that the relationship between handedness and mathematical ability is more complex and requires a polynomial function with a cubic and quartic term to be described properly.

Quartic functions can have up to five maxima and minima (three relative, and two at the extremes of the domain), be both monotonic and non-monotonic, and reduce into smaller-degree functions if necessary (by attributing the value 0 to one or more coefficients). For these reasons, quartic functions may be able to detect patterns of the relationship between handedness and mathematical ability impossible to identify with categorical measures or quadratic functions. Thus, our hypothesis is that including cubic and quartic terms in polynomial functions substantially contributes to the amount of variance in mathematical ability accounted for by handedness.

### Procedure

We ran five experiments differing from each other regarding the age of the participants and the type of mathematical skills assessed (e.g., arithmetic, reasoning). The aims of these experiments were (a) to evaluate which of the models introduced above best describes the relationship between handedness and mathematical ability; (b) to investigate the mediating effect of age, gender, and type of task on the link between handedness and mathematical ability; and (c) to quantitatively assess the effect of handedness on mathematical ability by calculating the percentage of variance (*R*^2^) of the participants' scores in mathematics explained by handedness, using 4th-degree polynomials.

The participants, aged 6–17, were recruited in Italian schools, between December 2013 and June 2015. Most of the participants (~80–85%) were from Italian middle-class families, while the rest were from foreign families.^2^ All the participants spoke fluent Italian and were not diagnosed with any learning disability. Parental consent was obtained for all the participants.^3^ The participants were administered (a) a set of different tests assessing mathematical ability (one for each experiment), and (b) the 10-item version of the Edinburgh Handedness Inventory (EHI; Oldfield, 1971)^4^. EHI is a multiple-item questionnaire, and thus is more sensitive and reliable than categorical measures of hand preference (Johnston et al., 2009). EHI provides a continuous measure of handedness (*H*), which was calculated using the formula:

$$\begin{array}{l} H=\frac{R-L}{R+L} \end{array}$$

where *R* and *L* indicate the number of preferences for the right and left hand, respectively. The range of values is between −1, for extreme left-handedness, and +1, for extreme right-handedness. Importantly, the participants were not divided into groups according to their hand preference. In fact, categories (e.g., right-handers, left-handers, and mixed-handers) are always arbitrary to some extent and hence may cause difficulties for comparing outcomes.

### Data analysis

Since our data were nested (i.e., most of the participants were recruited from different schools), a multi-level linear modeling (Goldstein, 2011) approach was applied to control for possible confounding effects (e.g., Type I error) due to the school of provenance of the participants. As noticed by several authors (e.g., Cheyne et al., 2010; Nicholls et al., 2010), the relationship between handedness and academic skills is not necessarily linear. Therefore, preliminarily to building the models, a series of linear regression analyses (method backward) was performed with *H, H* quadratic (*H*^2^), cubic (*H*^3^), and quartic (*H*^4^) functions as possible predictors, to look for potential non-linear (i.e., polynomial) relationships between *H* and scores in mathematics.^5^ Then, the functions of *H* [i.e., *F(H)*] calculated by the linear regression analysis were inserted—along with the participants' age and gender—into the models as independent variables. For each experiment, three multi-level linear models were run and compared to each other: the intercept model, the model including all the independent variables except the functions of handedness, and the model including all the independent variables. Finally, two additional regression analyses were performed (with predictors *H* and its functions) for males and females separately, in order to investigate possible gender differences in the relationship between handedness and mathematical ability.

## Experiment 1

In this experiment, we investigated the relationship between handedness and mathematical precocity in a sample of children aged 7–9. To evaluate whether Benbow's (1986, 1988) hypothesis—i.e., left-handers tend to be more precocious in mathematics—generalizes to the general population of third and fourth graders, we used items designed to assess the mathematical ability of 15-year-old students.

### Method

#### Participants

A total of 413 third and fourth graders (187 males, 226 females) with a mean age of 8.32 (*SD* = 0.62) years took part in this experiment only. The participants were recruited from five different schools in Italy.

#### Procedure

Along with the EHI, the participants were administered a set of seven OECD-Pisa items (OECD, 2012) assessing mathematical skill (score range 0–7). These items require the student to infer the correct solution from a given set of data and hence involve mathematical reasoning ability. In all the seven items, the participants were asked to choose one of the five possible answers.

The OECD-Pisa items are designed for students aged 15. Therefore, we expected the children to perform relatively poorly. Nonetheless, the contents of the selected items were manageable for children of third and fourth grades (e.g., problems involving only whole numbers, operations of addition and subtraction, and simple geometry), and the instructions were easily understandable.

### Results

**Figure 1:**
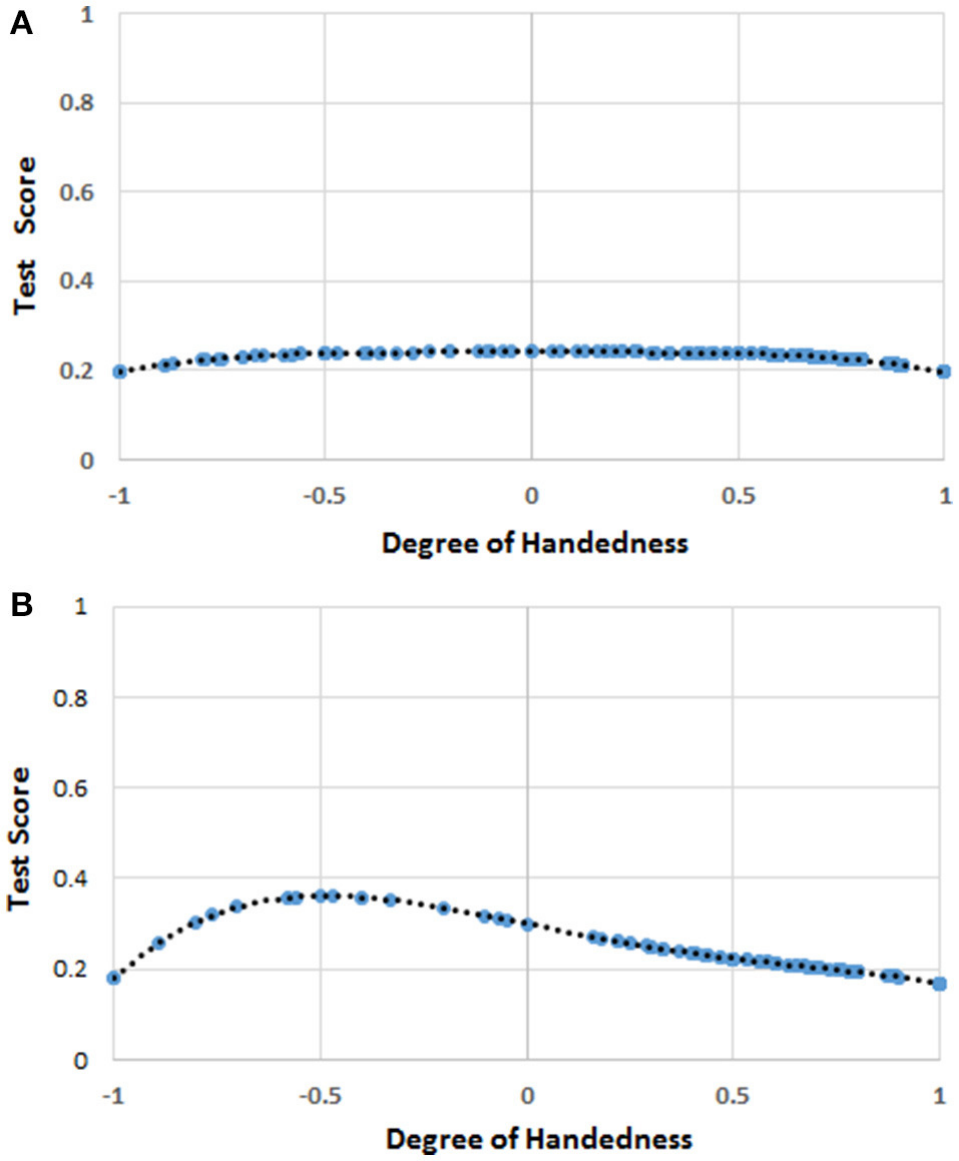
**(A)** The function [*F(H)*] of handedness (*H*) correlating with the score of mathematics in the whole sample. The blue circles represent *F(H)* values for the *H* values for which there were human observations. The black dots represent the trend line. The values on the y-axis were normalized by dividing *F(H)* by the possible maximum score (i.e., 7) on the test of mathematical ability. **(B)** The function [*F(H)*] of handedness (*H*) correlating with the score of mathematics in the female sample. The blue circles represent *F(H)* values for the *H* values for which there were human observations. The black dots represent the trend line. The values on the y-axis were normalized by dividing *F(H)* by the possible maximum score (i.e., 7) on the test of mathematical ability.

The mean score for *H* was 0.584 (*SD* = 0.470), while the mean score for mathematics was 1.55 (*SD* = 1.16). The linear regression analysis showed that only the quartic function of *H* (i.e., *H*^4^) was significantly correlated to the scores in mathematics (*b*_4_ = −0.312, *t* = −2.073, *r* = 0.102, *R*^2^ = 0.010, *p* = 0.039; *intercept* = 1.684, *p* < 0.001). The quartic function can be appreciated in Figure 1A.

The multi-level linear models showed a significant effect of age, no effect of gender, and only a marginally significant (*p* < 0.10) effect of *H*^4^ (Table 1). The effect of the school of provenance (random factor) was not significant.

**Table 1:** Parameters, coefficients, and standard errors in the multilevel models of Experiment 1.

**Parameter**	**Model 1**	**Model 2**	**Model 3**
**FIXED EFFECTS**			
Intercept	1.555 (0.076)^***^	−2.298 (0.759)^**^	−2.092 (0.765)^**^
Age		0.449 (0.090)^***^	0.438 (0.090)^***^
Gender		0.175 (0.111)	0.169 (0.110)
H^4^^a^			−0.256 (0.145)^†^
**RANDOM PARAMETERS**			
Intercept (School)	0.014 (0.018)	0.026 (0.026)	0.025 (0.025)
−2^*^log likelihood	1,290.6	1,263.5	1,260.4

*** p < 0.001, Two tailed;

** p < 0.01, two tailed;

* p < 0.05, two tailed;

† *p < 0.10, two tailed*.

a *The intercept was not inserted in the model because it is superfluous*.

#### Gender analysis

Two linear regression—one for males and one for females—were performed. The analysis showed no predictor in males, whereas it did in females (*b*_1_ = −1.275, *b*_3_ = 1.242, *b*_4_ = −0.885, *t* = 2.547, *r* = 0.284, *R*^2^ = 0.081, *p* < 0.001; *intercept* = 2.102, *p* < 0.001). The following function

$$\begin{array}{l} F\left(H\right)=-1.275\;H+1.242\;H^{3}-0.885\;H^{4}+2.102 \end{array}$$

depicting the relationship between *H* and mathematics scores in females can be seen in Figure 1B.

### Discussion

The results of this experiment suggest a quartic relationship between handedness and mathematical ability, especially for females. Benbow's (1986) hypothesis that left-handedness is a predictor of precocity in mathematical reasoning ability is not supported. In fact, the results appear to suit more—marginally in the whole sample, and significantly in females—Annett's (2002) conception of the disadvantage of the extremes.

## Experiment 2

Experiment 1 showed a quartic relationship between *H* and scores in mathematics, especially in females. However, the low scores achieved by the participants, due to the difficulty of the mathematical tasks, may have hidden other potential relationships between the two variables (e.g., the same relationship in males as well).

In this experiment, we replaced the OECD-Pisa items with six items designed for assessing mathematical literacy in fourth graders, and hence more suitable for the participants. Thus, we investigated the relationship between handedness and mathematical reasoning ability in primary school children again, but not focusing on mathematical precocity.

### Method

#### Participants

A total of 300 (151 males, 149 females) third and fourth graders took part in this experiment only. The participants' mean age was 8.46 (*SD* = 0.67) years. The children were recruited from nine schools in northern Italy.

#### Procedure

The participants were administered the EHI and a test consisting of six items of IEA-TIMSS (Mullis and Martin, 2013) international survey assessing mathematical literacy in fourth graders. Similar to OECD-PISA, the items of the IEA-TIMSS survey require solving a mathematical problem from a given set of data. The participants have to select an option among four possible answers.

### Results

**Figure 2:**
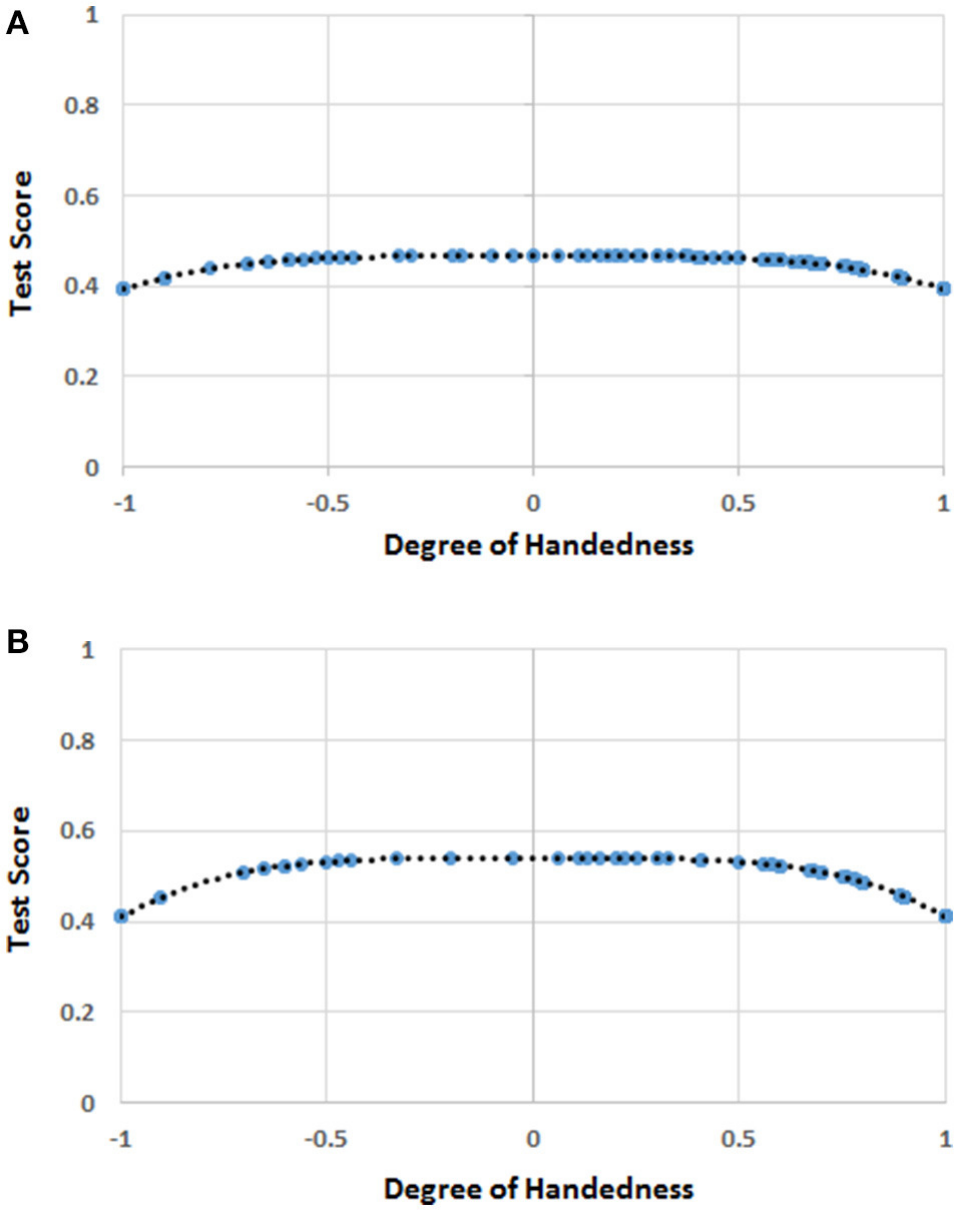
**(A)** The function [*F(H)*] of handedness (*H*) correlating with the score of mathematics in the whole sample. The blue circles represent *F(H)* values for the *H* values for which there were human observations. The black dots represent the trend line. The values on the y-axis were normalized by dividing *F(H)* by the possible maximum score (i.e., 6) on the test of mathematical ability. **(B)** The function [*F(H)*] of handedness (*H*) correlating with the score of mathematics in the sample of males. The blue circles represent *F(H)* values for the *H* values for which there were human observations. The black dots represent the trend line. The values on the y-axis were normalized by dividing *F(H)* by the possible maximum score (i.e., 6) on the test of mathematical ability.

The mean score for *H* was 0.614 (*SD* = 0*.5*29), while the mean score for mathematics was 2.57 (*SD* = 1.33). The regression analysis showed that only the quartic function of *H* (i.e., *H*^4^) was significantly correlated to the scores in mathematics (*b*_4_ = −0.438, *t* = −2.244, *r* = 0.130, *R*^2^ = 0.017, *p* = 0.026; *intercept* = 2.801, *p* < 0.001; Figure 2A).

The multi-level linear models showed a significant effect of gender, *H*^4^, and age (Table 2), while the effect of the school of provenance (random factor) was not significant.

**Table 2:** Parameters, coefficients, and standard errors in the multilevel models of Experiment 2.

**Parameter**	**Model 1**	**Model 2**	**Model 3**
**FIXED EFFECTS**			
Intercept	2.532 (0.105)^***^	−0.593 (0.948)	−0.172 (0.956)
Age		0.349 (0.113)^**^	0.325 (0.112)^**^
Gender		0.412 (0.149)^**^	0.448 (0.149)^**^
H^4^^a^			−0.447 (0.190)^*^
**RANDOM PARAMETERS**			
Intercept (School)	0.041 (0.043)	0.000^b^ (0.000)	0.000^b^ (0.000)
−2^*^log likelihood	1,018.5	1,002.1	996.7

*** p < 0.001, Two tailed;

** p < 0.01, two tailed;

* *p < 0.05, two tailed*.

a *The intercept was not inserted in the model because it is superfluous*.

b *The coefficient is set to 0 because it is redundant*.

#### Gender analysis

Regression analysis showed that *H*^4^ was still a predictor of the dependent variable (mathematics scores) for males (*b*_4_ = −0.779, *t* = −2.649, *r* = 0.212, *R*^2^ = 0.045, *p* = 0.009; *intercept* = 3.239, *p* < 0.001; Figure 2B), but not for females.

### Discussion

The results showed that the children at the two extremes of the distribution tended to achieve the poorest performance, but among males only. This outcome again supported Annett's (2002) conception of the disadvantage of the extremes. In this experiment too, gender moderated the effect that handedness exerted on the scores in mathematics. We will take up this issue in the General Discussion.

## Experiment 3

While the previous two experiments examined the effect of handedness on children's ability to solve mathematical tasks involving reasoning, this experiment evaluated the role of handedness on children's arithmetical ability. The used arithmetical tasks demanded only the knowledge and the application of simple algorithms (e.g., adding in column). Moreover, those who took part in this and the following two experiments were administered a mental rotation ability (MRA) task. Since MRA has been proposed as one possible link connecting handedness to mathematical ability (Annett and Manning, 1990; Casey et al., 1992), we tested whether the effect of handedness on arithmetical ability would remain significant even when MRA was controlled for.

### Method

#### Participants

One-hundred and sixty-two (78 males, 84 females) children took part in this experiment only. The participants were first, second, and third graders, and their mean age was 7.79 (*SD* = 0.89) years. The participants were recruited from one school in northern Italy.

#### Procedure

The participants were administered (a) the EHI,^6^ (b) a test of arithmetic, designed by the experimenters (score range 0–27), and (c) a 2-D mental rotation ability task suitable for children (score range 0–16; for details, see Cheng and Mix, 2014). In the test of arithmetic, the participants solved simple mathematical equations (e.g., 3 + 4 = ?) and missing-term problems (e.g., 3 +? = 7).

### Results

**Figure 3:**
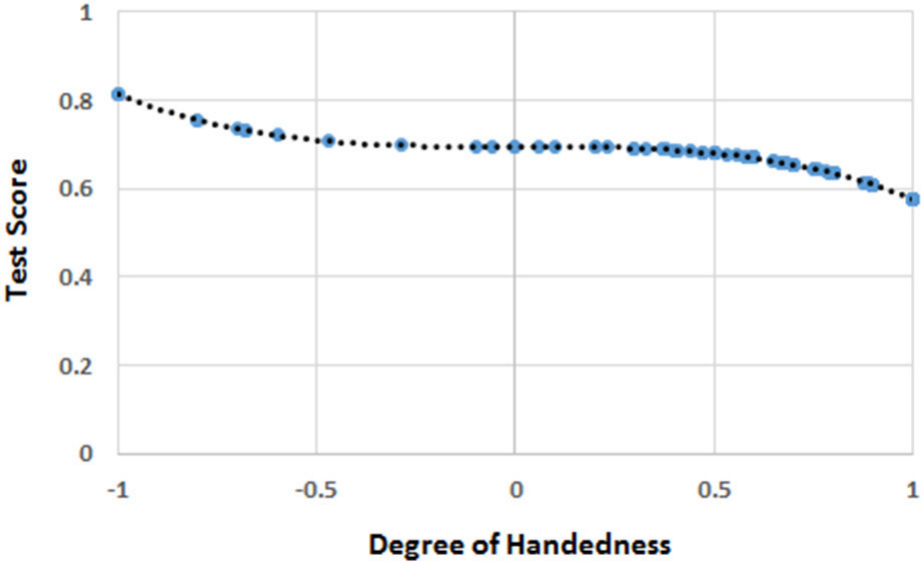
The function [*F(H)*] of handedness (*H*) correlating with the score of mathematics in the whole sample. The blue circles represent *F(H)* values for the *H* values for which there were human observations. The black dots represent the trend line. The values on the y-axis were normalized by dividing *F(H)* by the possible maximum score (i.e., 27) on the test of mathematical ability.

The mean score was 0.604 (*SD* = 0.473) for *H*, 17.43 (*SD* = 8.28) for the scores in arithmetic, and 13.65 (*SD* = 2.51) for mental rotation ability. The regression analysis showed that only the cubic function of *H* (i.e., *H*^3^) was significantly correlated to the scores in mathematics (*b*_3_ = −3.192, *t* = −2.192, *r* = 0.171, *R*^2^ = 0.029, *p* = 0.030; *intercept* = 18.759, *p* < 0.001), which can be appreciated in Figure 3.

The multi-level linear models showed a significant effect of *F*(*H*), mental rotation skills, and age, but no effect of gender (Table 3).

**Table 3:** Parameters, coefficients, and standard errors in the multilevel models of Experiment 3.

**Parameter**	**Model 1**	**Model 2**	**Model 3**
**FIXED EFFECTS**			
Intercept	17.426 (0.648)^***^	−44.883 (3.429)^***^	−43.353 (3.390)^***^
Age		7.083 (0.431)^***^	7.036 (0.421)^***^
Gender		0.818 (0.742)	0.839 (0.724)
MRA		0.543 (0.155)^***^	0.527 (0.151)^***^
H^3^^a^			−2.302 (0.811)^**^
**RANDOM PARAMETERS**			
Intercept (School)	0.000^b^ (0.000)	0.000^b^ (0.000)	0.000^b^ (0.000)
−2^*^log likelihood	1,143.6	958.1	950.3

*** p < 0.001, Two tailed;

** p < 0.01, two tailed;

* *p < 0.05, two tailed*.

a *The intercept was not inserted in the model because it is superfluous*.

b *The coefficient is set to 0 because it is redundant*.

Moreover, the analysis showed no significant correlation between *F*(*H*) and MRA scores (*r* = −0.053, *p* = 0.504).

#### Gender analysis

The regression analysis did not find any significant predictors either in males or females.

### Discussion

The results showed that handedness exerted a significant effect on the scores in mathematics even when MRA was controlled for. Interestingly, MRA and the function of handedness correlated with the scores in mathematics were not significantly correlated. This outcome suggests that the effects of handedness and MRA did not overlap. With respect to the shape of the relationship between handedness and arithmetical ability, the function showed a monotonic trend in favor of left-handers.

However, the relatively small number of participants may have been insufficient to detect potential alternative patterns among left-handers. Since left-handers were underrepresented compared to right-handers (as in the general population), the function might have fit the dependent variable regardless of the few left-handers of the sample. Put simply, the advantage of left-handers may have been due to a statistical artifact.

## Experiment 4

This experiment aimed at improving the design of the previous one by recruiting a larger sample. We thus wanted to test the advantage of left-handers in arithmetical tasks found in the previous experiment.

### Method

#### Participants

Seven-hundred and ninety-eight (417 males, 381 females) children took part in this experiment only. The participants were first, second, and third graders, and their mean age was 7.22 (*SD* = 0.91) years. The participants were recruited from six schools in northern Italy.

#### Procedure

Along with the EHI^7^ and the MRA task, the participants were administered a test of arithmetical abilities (AC-MT 6-11; Cornoldi et al., 2012). This test consisted of 26 items (score range 0–26) of basic arithmetic (e.g., addition, subtraction, multiplication, and identifying the greatest or the smallest number in a series).

### Results

**Figure 4:**
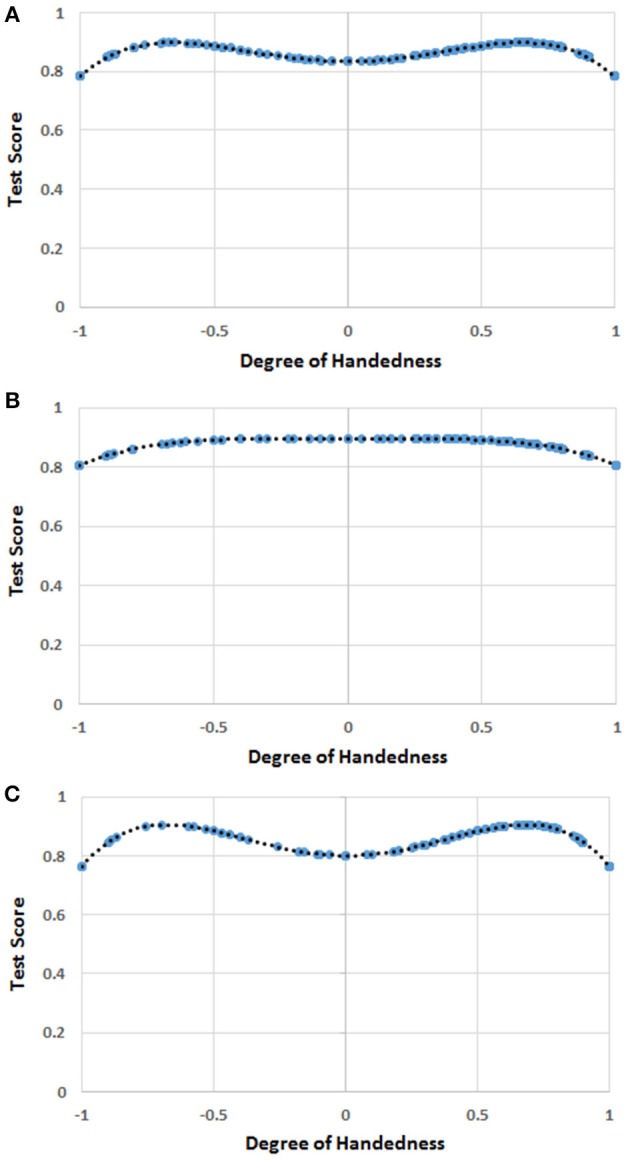
**(A)** The function [*F(H)*] of handedness (*H*) correlating with the score of mathematics in the whole sample. The blue circles represent *F(H)* values for the *H* values for which there were human observations. The black dots represent the trend line. The values on the y-axis were normalized by dividing *F(H)* by the possible maximum score (i.e., 26) on the test of mathematical ability. **(B)** The function [*F(H)*] of handedness (*H*) correlating with the score of mathematics in males. The blue circles represent *F(H)* values for the *H* values for which there were human observations. The black dots represent the trend line. The values on the y-axis were normalized by dividing *F(H)* by the possible maximum score (i.e., 26) on the test of mathematical ability. **(C)** The function [*F(H)*] of handedness (*H*) correlating with the score of mathematics in females. The blue circles represent *F(H)* values for the *H* values for which there were human observations. The black dots represent the trend line. The values on the y-axis were normalized by dividing *F(H)* by the possible maximum score (i.e., 26) on the test of mathematical ability.

The mean scores were 0.626 (*SD* = 0.522) for *H*, 21.81 (*SD* = 4.77) for arithmetical ability, and 13.04 (*SD* = 3.04) for mental rotation ability. The linear regression analysis showed that the quadratic (*H*^2^) and the quartic (*H*^4^) functions of handedness were correlated to the scores in the arithmetic test (*b*_2_ = 7.763, *b*_4_ = −9.083, *t* = 5.375, *r* = 0.260, *R*^2^ = 0.068, *p* < 0.001; *intercept* = 21.693, *p* < 0.001). We thus built the following function:

$$\begin{array}{l} F\left(H\right)=7.763\;H^{2}-9.083\;H^{4}+21.693 \end{array}$$

which is shown in Figure 4A.

The multi-level linear model showed a significant effect of *F*(*H*), MRA, and age, whereas no significant effect of gender was found (Table 4). The effect of the school of provenance (random factor) was not significant.

**Table 4:** Parameters, coefficients, and standard errors in the multilevel models of Experiment 4.

**Parameter**	**Model 1**	**Model 2**	**Model 3**
**FIXED EFFECTS**			
Intercept	22.023 (0.552)^***^	3.933 (1.321)^**^	5.187 (1.324)^***^
Age		1.561 (0.171)^***^	1.424 (0.171)^***^
Gender		0.477 (0.288)^†^	0.423 (0.284)
MRA		0.480 (0.049)^***^	0.456 (0.049)^***^
F(H)^a^			0.574 (0.118)^***^
**RANDOM PARAMETERS**			
Intercept (School)	1.573 (1.078)	0.873 (0.610)	0.802 (0.564)
−2^*^log likelihood	4,724.1	4,507.8	4,484.4

*** p < 0.001, Two tailed;

** p < 0.01, two tailed;

* p < 0.05, two tailed;

† *p < 0.10, two tailed*.

a *The intercept was not inserted in the model because it is superfluous*.

The correlation analysis showed that *F*(*H*) and MRA were correlated (*r* = 0.150, *p* < 0.001). We thus calculated the partial correlation—with the effect of the scores in MRA being partialled out—between *F*(*H*) and the scores in mathematics, and still found a significant correlation (*r* = 0.221, *R*^2^ = 0.049, *p* < 0.001).

#### Gender analysis

The regression analysis showed that *H*^4^ was a predictor (*b*_4_ = −2.346, *t* = −4.220, *r* = 0.203, *R*^2^ = 0.041, *p* < 0.001; *intercept* = 23.337, *p* < 0.001) of the dependent variable (mathematics scores) in males, while *H*^2^ and *H*^4^ were predictors (*b*_2_ = 11.788, *b*_4_ = −12.739, *t* = 4.542, *r* = 0.314, *R*^2^ = 0.098, *p* < 0.001; *intercept* = 20.824, *p* < 0.001) in females. The two functions are shown in Figures 4B,C.

No correlation was found between MRA scores and the quartic function in males. By contrast, a significant correlation was found between MRA scores and *F(H)* in females (*r* = 0.271, *p* < 0.001). We thus calculated the correlation between the females' scores in mathematics and *F(H)* with the scores in MRA being partialled out. This partial correlation was still significant (*r* = 0.234, *p* < 0.001).

### Discussion

The results of this experiment revealed once again that the participants occupying the two extremes of the handedness distribution achieved the worst scores in mathematics, and that the pattern obtained in the previous experiment (i.e., an advantage for left-handers over right-handers) was probably a statistical artifact. Moreover, the mixed-handed children—the ones in the center of the distribution—also obtained a relatively poor performance, especially among females. The latter outcome lends some support to the idea that mixed-handers are disadvantaged in mathematical abilities due to their hemispherical indecision (Crow et al., 1998; Cheyne et al., 2010). Interestingly, the results show an M-shaped pattern—indicating the inferior performance of the strong right- and left-handers, and of the mixed-handers—similar to the one found for mental rotation ability in Peters et al. (2006).

The shape of the relationship between handedness and scores in arithmetical ability does not differ substantially between genders. In fact, strong right- and left-handers achieved the worst results both in males and females, and the fact that mixed-handers do not seem to underperform among males might only be due to lack of statistical power. However, handedness appears to exert a greater influence on females' than does on males' arithmetical ability (*R*^2^ = 0.098 and *R*^2^ = 0.041, respectively).

## Experiment 5

The previous experiments tested the role of handedness in affecting children's mathematical ability. This experiment dealt with the relationship between handedness and high-school students' mathematical ability. The aim of this experiment was to examine whether handedness maintains a significant effect also on adolescents' performance in mathematics.

### Method

#### Participants

A total of 641 (211 males, 430 females) youngsters (aged 14–17) took part in this experiment only. The participants were ninth and tenth graders, and their mean age was 14.71 (*SD* = 0.76) years. The participants were recruited from three high schools^8^ in northern Italy.

#### Procedure

The participants were administered (a) the EHI, (b) a set of 10 OECD-Pisa items (OECD, 2012; score range 0–10) to assess mathematical ability, and (c) a revised version of Vandenberg and Kuse's (1978) 3-D mental rotation task assessing MRA (Version A; Peters et al., 1995; score range 0–24).

### Results

**Figure 5:**
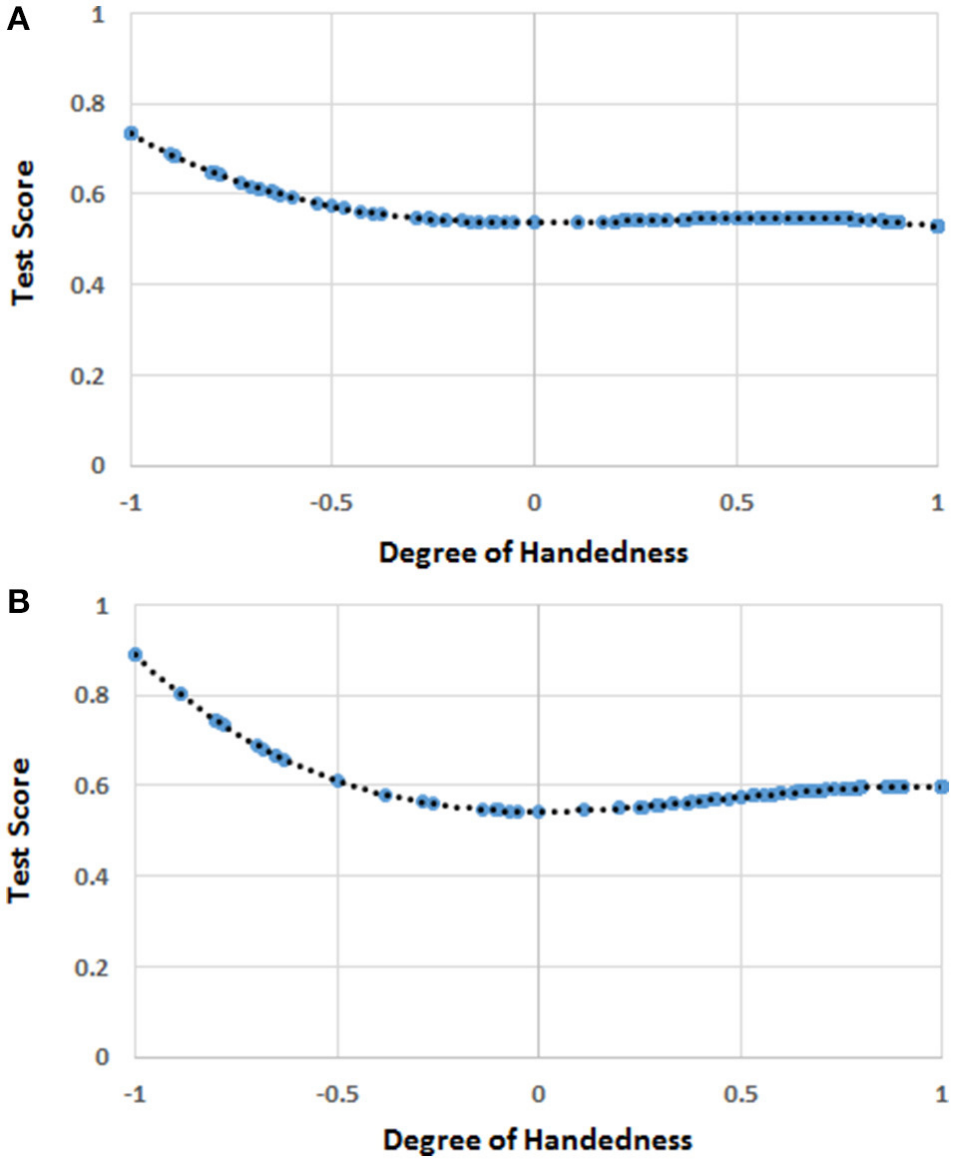
**(A)** The function [*F(H)*] of handedness (*H*) correlating with the score of mathematics in the whole sample. The blue circles represent *F(H)* values for the *H* values for which there were human observations. The black dots represent the trend line. The values on the y-axis were normalized by dividing *F(H)* by the possible maximum score (i.e., 10) on the test of mathematical ability. **(B)** The function [*F(H)*] of handedness (*H*) correlating with the score of mathematics in the sample of males. The blue circles represent *F(H)* values for the *H* values for which there were human observations. The black dots represent the trend line. The values on the y-axis were normalized by dividing *F(H)* by the possible maximum score (i.e., 10) on the test of mathematical ability.

The mean scores were 0.655 (*SD* = 0.432) for *H*, 5.46 (*SD* = 2.03) for mathematical ability, and 9.16 (*SD* = 5.03) for MRA. The linear regression analysis showed that the quadratic (*H*^2^) and the cubic (*H*^3^) functions of handedness were correlated to the participants' scores in mathematics (*b*_2_ = 0.934, *b*_3_ = −1.024, *t* = 2.524, *r* = 0.140, *R*^2^ = 0.020, *p* = 0.002; *intercept* = 5.369, *p* < 0.001). We thus built the following function:

$$\begin{array}{l} F\left(H\right)=\;0.934\;H^{2}-1.024\;H^{3}+5.369 \end{array}$$

which can be appreciated in Figure 5A.

The model showed a significant effect of *F*(*H*), scores in MRA, and age, whereas no significant effect of gender was found (Table 5). The effect of the school of provenance (random factor) was not significant.

**Table 5:** Parameters, coefficients, and standard errors in the multilevel models of Experiment 5.

**Parameter**	**Model 1**	**Model 2**	**Model 3**
**FIXED EFFECTS**			
Intercept	5.513 (0.497)^**^	0.225 (1.467)	0.101 (1.461)
Age		0.291 (0.094)^**^	0.296 (0.094)^**^
Gender		−0.229 (0.164)	−0.240 (0.164)
MRA		0.118 (0.015)^***^	0.118 (0.015)^***^
F(H)^a^			0.551 (0.245)^*^
**RANDOM PARAMETERS**			
Intercept (School)	0.724 (0.604)	0.598 (0.503)	0.564 (0.475)
−2^*^log likelihood	2,610.4	2,540.1	2,535.1

*** p < 0.001, Two tailed;

** p < 0.01, two tailed;

* *p < 0.05, two tailed*.

a *The intercept was not inserted in the model because it is superfluous*.

The correlation analysis showed no significant correlation between *F*(*H*) and MRA scores (*r* = −0.050, *p* = 0.207).

#### Gender analysis

The linear regression analysis showed that *H*^2^ and *H*^3^ were significant predictors (*b*_2_ = 1.968, *b*_3_ = −1.436, *t* = 2.625, *r* = 0.249, *R*^2^ = 0.062, *p* = 0.001; *intercept* = 5.431, *p* < 0.001) of the dependent variable (mathematics scores) in males only (Figure 5B) whereas no effect of handedness was found in females.

### Discussion

Handedness seems to affect mathematical ability in adolescents too. Surprisingly, this time, the left-handed participants happened to achieve the best scores. Moreover, as shown in Figure 5A, moderate right-handers were slightly better than strong right-handers and mixed-handers. Finally, this pattern occurred in males only. These outcomes support Benbow's (1986) idea that left-handers tend to be more talented in mathematics and that this occurs primarily in males. It must be noticed that this pattern occurred in a sample of the general population, and not only among extremely talented students as in Benbow's (1986) study.

## General discussion

The results of the five experiments show a significant effect of handedness on the participants' mathematical ability. The two main hypotheses of this study are supported: (a) the link between handedness and mathematical ability is more complex than linear and quadratic, and requires a polynomial function with cubic and quartic terms; and (b) the shape of this relationship seems to be moderated by participants' age and gender and the type of mathematical tasks used. Thus, handedness appears to be one of the biological correlates of mathematical ability.

Several additional points must be highlighted. First, the relationship between handedness and students' mathematical ability seems to be neither linear nor monotonic. This outcome suggests that the mere comparison between right- and left-handers is inadequate for describing how handedness and mathematical ability interact. It is also worth noting that non-linearity occurs in other well-known psychological phenomena, such as decision making (e.g., Hick's law; Hick, 1952), rate of forgetting (Sikström, 2002), and power of learning (Anderson et al., 1999). Second, the percentage of variance of scores in mathematics explained by handedness was between 3% and 10%. This amount is larger than the 1% previously reported for mathematical ability (Cheyne et al., 2010), overall cognitive ability (Nicholls et al., 2010) and mental rotation ability (Peters et al., 2006). Probably, this finding is due to the use of the cubic and quartic functions of the continuous measure of *H* (i.e., *H*^3^ and *H*^4^), which fit the data better than the simple linear and quadratic functions of *H* (i.e., *H* and *H*^2^), or the categorical measure of handedness. Third, handedness plays a significant role in the scores in mathematics even when the effect of MRA and age are controlled for. Moreover, even if MRA was a predictor of mathematical ability in experiments 3, 4, and 5, only a weak and often non-significant correlation was found between MRA and handedness functions. These considerations suggest that the relationship between handedness and mathematical ability is only partially mediated by MRA. Possibly, in line with Johnston et al. (2009) and Nicholls et al. (2010), the influence of handedness on mathematical ability reflects the effects of handedness on overall cognitive ability, rather than a specific one. Finally, the participants' gender and age, and the type of mathematical task influenced the shape of the relationship between handedness and mathematical ability. This result leads us to think that the predictions of the four models described in the Introduction are not necessarily mutually exclusive.

### The results in the light of the four models

The overall results seem to suit best Annett's (1985) conception of the heterozygous advantage. In Experiments 1, 2, and 4, strongly right- and left-handed children underperformed with respect to the rest of the sample. Interestingly, our results uphold Annett and Manning's (1989, 1990) concept of the disadvantage of dextrality. According to this hypothesis, strong right-handers—but not strong left-handers—are more likely to suffer from a deficit in spatial ability and, consequently, mathematical ability. In fact, in all the five experiments, the strong right-handed individuals showed a poorer performance compared to most (if not all) their peers.

Nonetheless, it seems reasonable that mathematical ability in children is based on their overall level of cognitive skill, and not only on their spatial ability. For example, strongly left-handed individuals may be more likely to suffer from some language-related deficits (Annett, 1985) due to an increased development of the right-hemisphere at the expense of the left-hemisphere (Geschwind and Galaburda, 1985).^9^ This possible language impairment may, in turn, affect negatively mathematical performance (e.g., difficulties in the comprehension of the instructions and terms of the task). Moreover, this pattern—i.e., strong right- and left-handers underperforming in mathematical ability—is also in line with Nicholls et al.'s (2010) concept of the disadvantage of the extremes in overall cognitive ability.

Benbow's (1986) hypothesis of the advantage of left-handers finds some support in our results too. While Experiments 1, 2, and 4 did not show any clear advantage for left-handers compared to right-handers, Experiments 3 and 5 did. Even assuming that the results of Experiment 3 concerning left-handers were not reliable because of the small number of left-handed participants, Experiment 5 strongly supported Benbow's hypothesis. In fact, consistent with Benbow (1986, 1988), the advantage of left-handers in mathematical ability concerned tasks involving reasoning, and occurred in males only. However, it is yet to be explained why the left-handers' advantage occurred only among high-school students, while the left-handed children's mathematical ability in tasks involving reasoning was not superior to the right-handers' one. Possibly, as proposed by Noroozian et al. (2002), left-handers may be considered as a heterogeneous group, consisting in part of gifted individuals, in part of typically developing individuals, and in part of underachievers. When individuals are tested in contexts of relative excellence—such as colleges or, in our case, liceos—the advantage of left-handers can occur, because those left-handers who suffer from any cognitive deficit are not likely to be in those contexts due to self-exclusion or academic selection. This explanation relies on the belief that both gifted and people with below-average cognitive ability are overrepresented among left-handers, in line with Benbow (1986) and Johnston et al. (2009, 2013), respectively. In other words, the alleged cause of left-handedness—superior development of the right-hemisphere (Geschwind and Galaburda, 1987) or pre- or peri-natal brain damage (Satz et al., 1985)—predicts when left-handedness is a correlate of giftedness or a disadvantage for cognitive ability.

However, the outcomes of the present study showed little evidence of the disadvantage of left-handers. The overall results seem to suggest a substantial equality between left- and right-handers—at least among primary school children. Possibly, the sample used in this study is too small to detect tiny differences between left- and right-handers. In fact, in Johnston et al. (2013), the difference in mathematical ability between left-handers and right-handers—in favor of the latter—barely reached the statistical significance in a sample of more than 5,000 children. Thus, the disadvantage of left-handers in mathematical ability, if any, may be extremely limited in size, and hence hard to detect. Another possible explanation is that, since Johnston et al. (2009, 2013) assessed handedness by considering only the participants' preferred writing hand, the disadvantage of left-handedness occurs when writing hand preference is considered. Conversely, when handedness is assessed as a continuous variable, the effect disappears.

Finally, the results support, at least to some extent, the hemispheric indecision hypothesis (Crow et al., 1998) as well. While the first three experiments did not offer any evidence of a disadvantage of mixed-handedness, in Experiment 4 and 5, mixed-handers showed a relatively poor performance in mathematical ability. Probably, Experiment 1, 2, and 3 did not have sufficient statistical power to identify a possible disadvantage of the mixed-handers, whereas the two experiments with the largest samples—i.e., Experiment 4 (*N* = 798) and 5 (*N* = 641)—did.

In a broader perspective, these results are in line with more recent research suggesting that the relationship between handedness and cognition is far too complex to be accounted for by simple models. For example, handedness appears to be a polygenetic—rather than monogenetic (e.g., Annett, 2002)—trait (Ocklenburg et al., 2013). It is thus reasonable to think that lateralization patterns are more complex than the right-left (or right-non-right) dichotomy may suggest (for a review, see Badzakova et al., 2016).

### Gender effects

Gender was a significant moderator of the relationship between handedness and mathematical problem-solving reasoning (Experiments 1, 2, and 5), whereas no effect was observed with arithmetical ability (Experiments 3 and 4). Interestingly, while in Experiment 1 handedness affected the female participants, in Experiment 2 and 5 only males' mathematics scores were related to handedness.

Providing an explanation of this pattern showing an interaction between handedness, gender, and type of mathematical ability is not simple. While the pattern occurred in females in Experiment 1, in Experiments 2, and 5 it did in males only. Regrettably, this is an outcome hardly accountable for by any of the four models. A possible explanation is that, unlike males, females tend to use verbal rather than spatial strategies when solving mathematical problems (Pezaris and Casey, 1991). Thus, the effects of handedness on performance in mathematical reasoning tasks—which usually involve spatial reasoning—may be less evident in females. Nonetheless, the fact that, in Experiment 1, only females' scores in mathematics were influenced by handedness does not fit the above hypothesis. Possibly, this empirical anomaly was due to the particular features of the design (e.g., a test for 15-year-olds administered to primary school children).

### Conclusions and recommendation for future research

The present study had two main aims: (a) evaluating the relationship between handedness and mathematical ability by using quartic functions of *H*-values; and (b) exploring the role of the moderating effects of age, gender, and type of mathematical task on this relationship. The results showed several significant interactions between the above variables—along with a nearly generalized tendency toward the disadvantage of the extremes. Moreover, the amount of variance (*R*^2^) in mathematics performance accounted for by handedness was approximately between 3 and 10%, and remained significant even when the effects of participants' mental rotation ability (MRA) were partialled out. Such high percentages were probably the consequence of using cubic and quartic functions, which fit the pattern of data better than linear and quadratic functions.

These outcomes have two important consequences. First, the different predictions of the four models on the role of handedness on cognitive and mathematical abilities are not necessarily irreconcilable. The present study, however, represents only a first step toward the development of a more comprehensive model. Second, the size of the effects of handedness on mathematical ability seems to be more relevant (up to 10% of the variance) than it has been proposed so far (1%). Thus, handedness cannot be considered a negligible predictor of mathematical ability.

Further research is needed to draw a more precise causal model of the effect of handedness on cognition and mathematical ability. We thus propose some recommendations for extending the design of the present study. First, we found that mental rotation ability was weakly correlated with the polynomial functions of handedness predicting the scores in mathematics. Examining the relationship between handedness and a broader range of measures of cognitive ability (e.g., fluid intelligence, working memory, and phonological processing) is thus necessary to find a “cognitive link” between handedness and mathematical ability. For example, the use of path analysis and latent-factor analysis may enable us to find more complex systems of relationships between handedness, cognitive abilities, and academic skills such as mathematics. Second, future studies should include participants of different ages (e.g., younger than six, older than 17), to have a more comprehensive view of the effects of handedness on cognitive and mathematical skills during development. Third, gender seems to affect the relationship between handedness and mathematical problem-solving ability significantly. For this reason, future investigations should control for gender differences in the strategies adopted when solving mathematical tasks involving reasoning. Fourth, the type of mathematical task appears to be another moderating variable. Therefore, testing the same participants on both arithmetical and problem-solving skills would help to evaluate better the role played by this variable on the relationship between handedness and mathematical ability. Moreover, the research on the topic should go beyond academic measures of mathematical ability—like the ones used in the current investigation—and be extended to numerical cognition (Feigenson et al., 2004; Reyna et al., 2009; Peters, 2012) and its components (e.g., Peters and Bjalkebring, 2015). For example, it has been found that the approximate number sense correlates with measures of mathematical literacy (Libertus et al., 2011). It is thus important to investigate whether the relationship between handedness and numerical cognition presents the same features reported in the current study (e.g., non-linearity and influence of the type of task, age, and gender). In a broader perspective, including measures of cognitive ability (e.g., fluid intelligence and working memory capacity) to draw more complex systems of relationships between handedness, cognition, and mathematical ability should be a priority in the field. Finally, using measures of hand skill (e.g., square checking task)—along with questionnaires assessing hand preference in everyday life activities (e.g., Annett, 1970; Oldfield, 1971)—would help test whether the shape of the relationship between handedness and mathematical ability varies according to how handedness is assessed.

## Ethics statement

The principals of the schools involved provided written permission for the administration of the tests. Parental consent was asked and obtained for all the participants. Individuals with any diagnosed learning disability were excluded.

## Author contributions

GS and FG developed the study concept. MS, GB, and MB collected the data. GS performed the analyses of the data. All the authors contributed to the drafting of the paper.

### Conflict of interest statement

The authors declare that the research was conducted in the absence of any commercial or financial relationships that could be construed as a potential conflict of interest.

## Footnotes

^1^It is worth mentioning that there is not a perfect correspondence between handedness and lateralization of cognitive function. For example, only about 40% of left-handers have their motor speech area located in the right-hemisphere (Gutwinski et al., 2011). Analogously, only one third of the people with more developed right hemisphere are not right-handed (Geschwind and Behan, 1984).

^2^Due to restrictions related to Italian privacy laws, we could not collect any information about parental income and countries of provenance of the participants. The large majority of the participants with foreign origins consisted of children from Eastern Europe and Northern Africa who were born or spent most of their lives in Italy. With regard to parental income, Johnston et al. (2009) reported that handedness was not related to socioeconomic characteristics in a large sample of children. Thus, there is no reason to think that socio-economic status may be a confounding variable for the present study.

^3^Most of the parents provided the school with written and informed consent. In some cases, the schools had previous official agreements with the parents regarding the type (e.g., paper and-pencil tests) of permitted activities their children could engage in. In the remaining cases, the schools adopted other customary practices (e.g., opt-out consent). This process was entirely led by the principals (who are the legal representatives of the schools) or a delegate teacher. This study was not required to be reviewed and approved by an ethics committee according to the national and institutional requirements for this type of research.

^4^The item “striking a match” was considered inappropriate and obsolete for the participants, and thus with the item “dealing cards” (Groen et al., 2013).

^5^It must be mentioned that the current investigation does not directly address the issues regarding the dichotomy between direction (i.e., positive or negative EHI score) and degree (i.e., how far from zero the EHI score is) of handedness (for an outlook on this debate, see Prichard et al., 2013). Additional research is needed to evaluate the role of the direction and degree of handedness in affecting cognitive/academic skills.

^6^The youngest participants (i.e., first graders; 6- and 7-year-olds) were read the instructions of the questionnaire. When necessary, the testers explained the inquired manual act and asked the children to mimic it.

^7^Like in Experiment 3, the first-graders were assisted in the administration of the EHI (see footnote 5).

^8^The schools were labeled as *liceo* according to the Italian school system. Liceos are high schools whose curricula are designed to prepare students for college.

^9^It is worth noting that more recent studies have suggested that the right hemisphere also contributes to language comprehension and production (for a review, see Poeppel et al., 2012). Nonetheless, the primary role of the left hemisphere in such functions has been convincingly established (e.g., Turken and Dronkers, 2011).
